# Molecular pathogenesis: Connections between viral hepatitis-induced and non-alcoholic steatohepatitis-induced hepatocellular carcinoma

**DOI:** 10.3389/fimmu.2022.984728

**Published:** 2022-09-15

**Authors:** Zelin Tian, Chen Xu, Peijun Yang, Zhibin Lin, Wenlong Wu, Wenjie Zhang, Jian Ding, Rui Ding, Xuan Zhang, Kefeng Dou

**Affiliations:** ^1^ Department of Hepatobiliary Surgery, Xijing Hospital, Air Force Medical University, Xi’an, China; ^2^ Chinese Education Ministry’s Key Laboratory of Western Resources and Modern Biotechnology, Key Laboratory of Biotechnology Shaanxi Province, College of Life Sciences, Northwest University, Xi’an, China

**Keywords:** hepatocellular carcinoma, viral hepatitis, non-alcoholic steatohepatitis, molecular pathogenesis, HBV - hepatitis B virus, HCV (hepatitis C)

## Abstract

Hepatocellular carcinoma(HCC) is the sixth most common cancer in the world and is usually caused by viral hepatitis (HBV and HCV), alcoholic, and non-alcoholic fatty liver disease(NAFLD). Viral hepatitis accounts for 80% of HCC cases worldwide. In addition, With the increasing incidence of metabolic diseases, NAFLD is now the most common liver disease and a major risk factor for HCC in most developed countries. This review mainly described the specificity and similarity between the pathogenesis of viral hepatitis(HBV and HCV)-induced HCC and NAFLD-induced HCC. In general, viral hepatitis promotes HCC development mainly through specific encoded viral proteins. HBV can also exert its tumor-promoting mechanism by integrating into the host chromosome, while HCV cannot. Viral hepatitis-related HCC and NASH-related HCC differ in terms of genetic factors, and epigenetic modifications (DNA methylation, histone modifications, and microRNA effects). In addition, both of them can lead to HCC progression through abnormal lipid metabolism, persistent inflammatory response, immune and intestinal microbiome dysregulation.

## 1 Introduction

Hepatocellular carcinoma, accounting for 75-85% of primary liver cancer cases, is the sixth most common cancer and the third leading cause of cancer death globally in 2020, with approximately 906,000 new cases and 830,000 deaths annually (1). Patients with HCC are usually asymptomatic in the early stage and are often in the advanced stage when they have typical symptoms, such as liver pain, jaundice, ascites, and liver failure (2). Common treatments for HCC include radiofrequency ablation(RFA), hepatic resection, liver transplantation, transcatheter arterial chemoembolization(TACE), tyrosine-kinase inhibitors (sorafenib), radiotherapy, and immune oncology (2, 3). Hepatitis B virus (HBV) or hepatitis C virus (HCV) infection, alcohol abuse, and NAFLD are the most common risk factors for HCC (2, 4–6).

Although the burden of nonalcoholic steatohepatitis (NASH)-induced HCC is increasing, chronic viral hepatitis (HBV and HCV) remains the leading cause of HCC, causing 80% of cases worldwide (5, 6). Liver cirrhosis caused by chronic HBV or HCV infection is an important factor in the development of HCC. It is worth noting that HCC can also appear in patients with chronic viral hepatitis infection without cirrhosis (7). Patients with viral hepatitis often have co-infections. Chronic hepatitis B (CHB) infection affected approximately 257 million people worldwide, of which 48-60 million were co-infected with HDV and 2.6 million were co-infected with HCV (8). Individuals co-infected with HBV/HCV have an increased incidence of HCC and a poorer prognosis compared with HBV or HCV mono-infection (2, 9). In the progression of CHB to HCC, synergistic risk factors include male sex, alcohol abuse, high viral load(HBV DNA > 10^6^ U/mL), HBV genotype C, presence of cirrhosis, and hepatitis B e-antigen positivity (10, 11). The main treatment for CHB is the use of nucleotide analogues (NA) to inhibit HBV replication (1, 12). HCV infection is another major cause of chronic liver disease. Additional risk factors that may increase the risk of HCC in patients with HCV infection include male sex, diabetes and obesity, alcohol abuse, and HCV genotype 3 (6, 13). The development of direct-acting antiviral therapy (DAA) has improved the prognosis of HCV-induced HCC, and achieving sustained virologic response (i.e., virological cure, SVR) is associated with a significant reduction in HCC risk (14, 15).

With the increasing incidence of metabolic diseases such as diabetes and obesity, NAFLD has become an increasingly serious health problem (16). NAFLD can develop into NASH, liver fibrosis, liver cirrhosis, and eventually HCC. In recent years, with the widespread vaccination of the hepatitis B vaccine and the popularization of anti-HBV and HCV treatment, the incidence of virus-induced HCC has steadily decreased. NAFLD/NASH has gradually developed into one of the main causes of HCC in developed countries (16, 17). Patients with NAFLD have a very low risk of progressing to cirrhosis, but patients with NASH have a significantly increased risk of progressing to cirrhosis and even HCC. Compared with viral hepatitis-related HCC, NASH-related HCC patients tend to be older, have a better liver function, larger tumor size, and longer overall survival(OS) (18). In addition, some patients can directly progress to HCC without cirrhosis, and these patients always have a worse prognosis (19).

In this review, we discuss the similarities and differences in the molecular mechanisms of viral hepatitis and NASH-induced HCC. We accept that this review will help clinicians in the diagnosis and treatment of HCC patients, and provide guidance for the development of new molecular therapeutic targets and therapeutic drugs.

## 2 HBV-specific induced HCC

### 2.1 General features of the HBV

**Figure 1:**
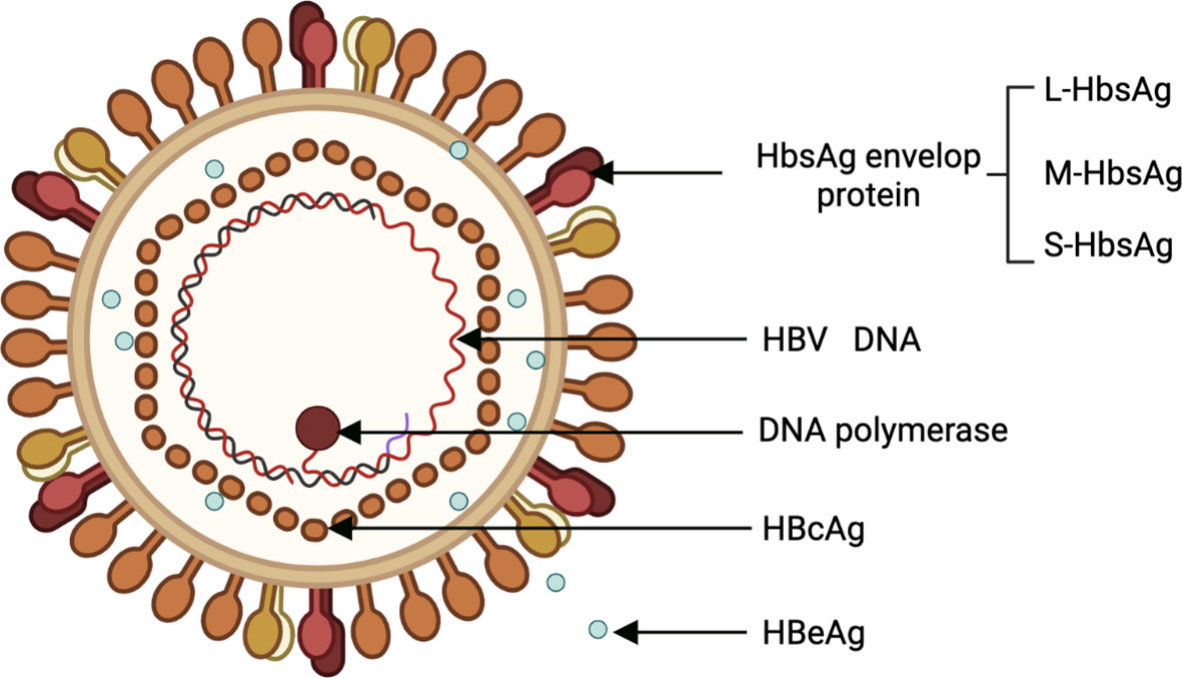
Structural of HBV. HBV, Hepatitis B virus.

HBV is a para-retrovirus that was discovered by American geneticist Baruch Blumberg in 1965. Its genome is a 3.2-kb double-stranded loop of DNA (20). HBV has 10 genotypes (A to J), with genotypes C, B, F, D, and A are associated with the development of HCC (6). The HBV genome consists of 4 overlapping open reading frames (ORFs): Pre-S/S, X, P, and pre-C/C, which are transcribed to produce 5 messenger RNAs (mRNAs) (21). Viral protein products include 3 surface proteins (also known as large/pre-S1 (L-HBsAg), medium/pre-S2 (M-HBsAg), and small/major (S-HBsAg)), the excreted “e” antigen (HBeAg), the core antigen (HBcAg), the X protein (HBx), and the viral polymerase (DNA polymerase, reverse transcriptase, and RNaseH activity) (**Figure 1**). HBx is required for HBV replication and plays an important role in both HBV and HBV-induced HCC progression (21).

### 2.2 HBV DNA integration in host chromosomes

HBV DNA integration into the host chromosome is not an essential step in the HBV life cycle (22). However, this phenomenon can lead to the instability of the host genome, insertional mutations in proto-oncogenes and tumor suppressor genes, which in turn promote the occurrence of HCC (23). HBV DNA integration was observed in approximately 80% of HBV-induced HCC patients, and the frequency of integration was significantly higher in tumor tissues than in adjacent tissues (24). Integration sites tend to be located near repeat regions, CpG islands, and telomeres, leading to chromosomal instability (24). There are many target genes affected by HBV genome integration, such as TERT, MLL4, CCNE1, MLL2, ARID1A, ARID1B, ARID2, MLL3, et al. (25–27). Integration of HBV DNA can also induce the persistent expression of mutated and truncated HBsAg, HBcAg, and HBx proteins. High expression rates of these proteins can promote HCC development through endoplasmic reticulum and mitochondrial stress responses (28, 29).

### 2.3 HBV-induced epigenetic dysregulation

Epigenetic changes include all chromatin changes, while DNA sequence does not change, and can be divided into three types: DNA methylation, histone modification, and RNA-related silencing.

#### 2.3.1 DNA methylation

In HBV-related HCC, DNA hypermethylation occurs at CpG islands in the promoter regions of specific tumor suppressor genes, resulting in the silencing of tumor suppressor genes, which in turn promotes the occurrence of HCC (30, 31). Persistent HBV infection can cause hypermethylation of p16^INK4A^, and HBx may play a key role in this process (32). RASSF1A (a cell cycle-related tumor suppressor protein) methylation occurs in more than 50% of HBV-infected livers, and its methylation level is also considerably increased early in the pathogenesis of HCC (33). CDH1 encoded a protein named epithelial cadherin (E-cadherin), which plays a crucial role in the epithelial-mesenchymal transition process. HBx can downregulate E-cadherin protein levels *via* promoting CDH1 hypermethylation (34). In contrast to hypermethylation, DNA hypomethylation is assumed to be a genome-wide event in HCC, which can lead to genomic instability. HBx can selectively promote regional hypermethylation of specific tumor suppressor genes by upregulating DNMT1, DNMT3A1, and DNMT3A2, and can also induce global hypomethylation of HSATII by downregulating DNMT3B (35).

#### 2.3.2 Histone modification

Histones can be reversibly modified by acetylation, methylation, phosphorylation, or ubiquitination. These modifications have implications for gene activation, gene repression, DNA repair, and cancer development (36). Experiments by Liu et al. demonstrated that HBx can promote the expression of IGF-II by inducing the hypomethylation of the P3 and P4 promoters in HCC cells and HCC specimens. HBx can bind to MBD2 and CBP/p300 to promote MBD2-HBx-CBP/p300 complex formation, which in turn promotes the acetylation of the corresponding histones H3 and H4, providing new insights into the pathogenesis of HBx-mediated HCC (37). Arzumanyan’s study revealed that HBx protein can promote epigenetic modulation of E-cadherin transcriptional activity through histone deacetylation and miR-373 (34). Histone H3 lysine 4 methyltransferase SMYD3 has been shown to promote the transcriptional activation of genes involved in the development of HCC, such as C-MYC, JAK/STAT3, CDK2, and MMP2 (38–40).

#### 2.3.3 MicroRNAs in HBV- HCC

MicroRNAs are small non-coding RNA with 19-25 nucleotides in length, which lead to gene silencing through translation inhibition or targeted degradation of mRNA. In recent years, more and more studies have shown that some MicroRNAs can be regulated by HBV infection and play a key role in hepatocarcinogenesis (41, 42). For example, miR-18a, miR-21, miR-221, miR-222, and miR-224 were upregulated in HBV-related HCC tissues, while miR-26a, miR-101, miR-122, miR-125b, miR-145, miR-199a, miR-199b, miR-200a, and miR-223 were upregulated in HBV-related HCC tissues. These miRNAs have been proved to affect HCC progression *via* targeting JAK/STAT, PI3K/MAPK, TP53, WNT/β-catenin pathways (41).

### 2.4 The role of HBV-encoded proteins in HCC

HBx protein plays an indispensable role in the life cycle of HBV and the progression of HCC. HBx plays its role mainly through the following four mechanisms: 1) HBx gene can be integrated into hepatocyte genome and affect genomic stability; 2) Induced epigenetic modifications such as DNA methylation, histone acetylation, and MicroRNA expression; 3) Oxidative stress induced by interaction with mitochondria and other proteins; 4) Participate in the regulation of proto-oncogene activation and tumor suppressor gene inactivation (8). The first two of these mechanisms have been described above. HBx is the most common open reading frame(ORF) integrated into the host genome in HBV-induced HCC specimens and the integrated HBx is frequently mutated (43, 44). And both of them appear to be important steps in HCC tumorigenesis (45, 46). HBx can trans-activate cellular promoters and enhancers and participate in the regulation of inflammatory proliferation-related signaling pathways, such as NF-κB, Ras/Raf mitogen-activated protein kinase (MAPK), c-Jun N-terminal kinase, Jak1/STAT, protein kinase C (PKC) and Src kinase, etc (47, 48). HBx in the cytoplasm can also bind to p53, prevent p53 nuclear localization, lead to dysregulation of cell cycle checkpoints, and inhibit p53-dependent apoptosis and DNA repair (49, 50).

HBx can also induce upregulation of vascular endothelial growth factor (VEGF) and angiogenic factor ang2(ANG2), stabilize HIF1α, and promote the angiogenesis of HCC (51, 52).

In addition, HBsAg can enhance the malignant potential of HBV-induced HCC by enhancing the IL-6-STAT3 pathway (53). HBV core protein can increase the production of cytokines and is related to the host immune response, both of which play a role in HBV-related HCC (54, 55).

### 2.5 Genetic variations in HBV

Due to the lack of proofreading function of HBV reverse transcriptase, the replication error rate of HBV DNA is much higher than that of other DNA viruses. Among the three envelope protein forms of HBsAg (L-HBsAg, M-HBsAg, and S-HBsAg), the amino acid position between 99-169 of S-HBsAg is called the main hydrophilic region (MHR), and the antigenic cluster “A” is located in it. Mutations in MHR will affect HBsAg antigenicity and lead to vaccine-induced immune escape (56). Pres/S region mutations may give rise to endoplasmic reticulum stress, oxidative DNA damage, and genomic instability in hepatocytes (57). In addition, K130M/V131I double mutations in the X gene, A1762T/G1764A double mutations in the basic core promoter (BCP), and mutations in the reverse transcriptase region will increase the risk of HCC (58–61).

## 3 HCV-specific induced HCC

### 3.1 General features of HCV

**Figure 2:**
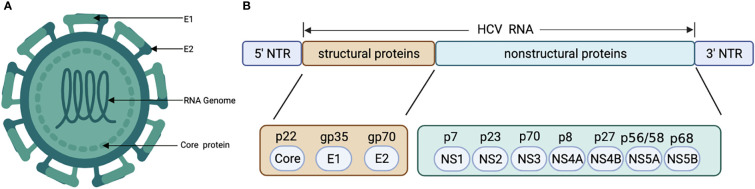
Structural **(A)** and genetic organization **(B)** of HCV. HCV, Hepatitis C virus.

HCV genome is a 9.6 kb positive-strand single-stranded RNA virus with highly conserved 5’ and 3’ untranslated regions, encoding 3 structural proteins (core, E1, E2) and 7 nonstructural proteins (p7, NS2, NS3, NS4A, NS4B, NS5A, and NS5B) (**Figure 2**) (62). Unlike HBV viruses, HCV cannot stably integrate into the host genome and requires continuous replication to gain viability (63). There are 6 major genotypes of HCV, and genotypes 3 and 6 infections have been reported to have a higher risk in hepatocarcinogenesis (13, 64).

### 3.2 HCV-induced epigenetic dysregulation

Unlike HBV, HCV is not integrated into the host genome, but can promote HCC progression through epigenetic dysregulation.

#### 3.2.1 DNA methylation

As with HBV-positive HCC, DNA methylation also plays an important role in HCV-positive HCC. The clinical study of Zekri showed that the progression of HCV-induced HCC was associated with increased DNA promoter methylation (65). SOCS-1, as a negative regulator of the JAK/STST pathway, often acts as a tumor suppressor gene (66). The methylation of SOCS-1 is more common in HCV-positive HCC (compared to HCV-negative HCC) (67). HCV protein can down-regulate Gadd45β expression by promoting hypermethylation of Gadd45β promoter, resulting in defective cell cycle arrest and leading to hepatocarcinogenesis (68). Duong’s study revealed that HCV could reduce the transcriptional activation of interferon- alpha(IFN-α) by promoting STAT1 and PP2Ac hypomethylation (69). In addition, Hypermethylation of APCαp15αp14αp73αp16αO^6^MGMT, and IGF2 can also affect the progression of HCV-induced HCC (30, 70, 71).

#### 3.2.2 Histone modification

Hamdane’s research exposed a paradigm that chronic HCV infection induces 27 histone 3 (H3K27Ac) acetylation modifications can promote hepatocarcinogenesis (72). In addition, acetylation of histone H3 lysine 9 (H3K9Ac) plays a similar role in HCV-mediated carcinogenesis (73). Histone demethylase member KDM5B/JARID1B can enhance HCV-induced HCC cell proliferation *via* regulating its downstream genes E2F1 and E2F2 (74). HCV core protein can induce dysregulation of HOX gene by impairing histone H2A mono-ubiquitination, which will promote HCC development (75).

#### 3.2.3 MicroRNAs in HCV-induced HCC

Studies have reported that specific liver and serum MicroRNAs are involved in the pathogenesis of HCV-induced HCC, including miR-193b, miR-155, miR-122, etc (76). In general, the mechanism of MicroRNAs in HCV-related HCC is not in-depth enough, and the number of related reports is smaller than that of HBV-related HCC reports.

### 3.3 The role of HCV-encoded proteins in HCC

There are 10 kinds of HCV gene products, among which the core, NS3, NS5A, and NS5B proteins potentiate carcinogenic pathways. They play an important role in promoting cell proliferation, regulating cytokines, oxidative stress, apoptosis, and HCV-related metabolic disorders and liver disease progression (77). HCV NS5B can form cytoplasmic complexes with Rb, leading to activation of E2F-dependent transcription and increased cell proliferation (78). HCV NS5A and NS3 can bind to P53 and down-regulate the expression of cell cycle regulation gene P21 (79, 80). P73 can interact with HCV core protein, leading to nuclear translocation of the core protein and promotes cell proliferation in a p53-dependent manner (81). HCV core protein can also induce oxidative DNA damage, enhance ROS production, and inhibit apoptosis (82).

### 3.4 Genetic variations in HCV

There are relatively few studies on the correlation between HCV characteristic mutations and HCC development (compared with HBV), among which HCV core gene mutations are the most studied. Studies have shown that HCV core A028C, G209A, C219U/A, U264C, A271C/U, C378U/A, G435A/C, and G481A mutations were significantly associated with increased HCC risk, while U303C/A mutation predicted reduced HCC risk (83).

## 4 NASH-induced HCC

### 4.1 Pathophysiology of NASH

The progression of NAFLD to NASH is a complex multi-factor process, whose detailed mechanism has not been fully elucidated. It is currently mainly accepted by the public as the “two-hit hypothesis” (84). The core idea is that liver steatosis and insulin resistance are the “first hit”, which leads to riglycerides accumulate in liver cells (85). Then, under the joint action of inflammatory factors, oxidative stress and endoplasmic reticulum stress, liver dysfunction such as hepatocyte inflammation, liver fibrosis and cirrhosis is developed, namely the “second hit” (86).

In recent years, there is a new consensus on the “multiple parallel hit hypothesis” to replace the “two-hit hypothesis”. The multiple parallel hit hypothesis suggested that NASH is the result of a combination of genetic differences, insulin resistance, lipid metabolism abnormalities, endoplasmic reticulum stress, mitochondrial dysfunction, and intestinal microbiota (87, 88).

### 4.2 Genetic factors

Genetic mutations in the protein-like phospholipase domain-containing protein 3 PNPLA3 gene is the most well-known mutations associated with NASH-related HCC progression (89, 90). PNPLA3 rs738409 c.444C>G minor allele (encoding the I148M variant) is associated with increased lipid accumulation and fibrosis in the liver. It also predisposes individuals to fatty liver-related diseases ranging from simple steatosis to steatohepatitis, NASH, and HCC (91). Overexpression of I148M PNPLA3 protein in mouse liver promoted steatosis by triggering metabolic reprogramming and driving inflammatory pathways (92).

17β-HSD13 is thought to be the pathogenic protein of NAFLD development (93). Chen et al. demonstrated that hydroxysteroid 17-Beta Dehydrogenase 13 (HSD17B13) was low expression in HCC and was associated with poor prognosis (94). HSD17B13 rs72613567 (a splice variant with an adenine insertion) reduced the risk of NASH and progressive liver injury (95).

In addition, TM6SF2 rs58542926 variant and MBOAT7 rs641738 variant have also been proved to be genetic variants susceptible to NAFLD-related HCC, and their effects are not necessarily mediated by the development of liver fibrosis (96–98).

### 4.3 NASH-induced epigenetic dysregulation

#### 4.3.1 DNA methylation

Epigenetic changes such as abnormal DNA methylation are considered to be an important mechanism for NASH progression. It induced gene silencing associated with DNA damage and repair, lipid and glucose metabolism, and fibrosis progression *via* enzyme methyltransferase (DNMT) (99). Kuramoto’s study further confirmed that NASH-specific DNA methylation change may be involved in the development of NASH-associated multistage HCC (100, 101).

#### 4.3.2 Histone modification

The histone deacetylase 8 (HDAC8) has been defined as a modifier of chromatin tissue in NASH-associated HCC (102, 103). HDAC8 can inhibit p53/P21 mediated apoptosis and stimulate β-catenin dependent cell proliferation. Knockdown of HDAC8 can reverse insulin resistance and reduce NAFLD-related tumorigenicity (103).

#### 4.3.3 MicroRNAs in NASH-induced HCC

Accumulating evidence has demonstrated the role of microRNAs in epigenetic dysregulation of metabolic processes in NAFLD, NASH, and HCC. Takaki’s study showed that silencing of miR-122 is an early event in NASH and may be a novel molecular marker for assessing HCC risk in NASH patients (104). In addition, miR-21, miR-29, miR-23, miR-155, miR-221, miR-222, miR-106, miR-93, and miR-519 have also been confirmed to be associated with carcinogenic effects associated with NASH (105).

## 5 Connections between viral hepatitis-induced and NASH-induced HCC

Viral hepatitis-induced and NASH-induced HCC have been described in detail in a large number of previous reviews, but there is no article to summarize and discuss them together. In the previous section, we have discussed the specific pathways of viral hepatitis-induced and NASH-induced HCC, respectively. In this section, we will describe the common mechanism of them, and we will select the most important research hotspots for description, including metabolic pathways, inflammatory pathways, and intestinal microbiota dysfunction.

### 5.1 Metabolic pathways

**Figure 3:**
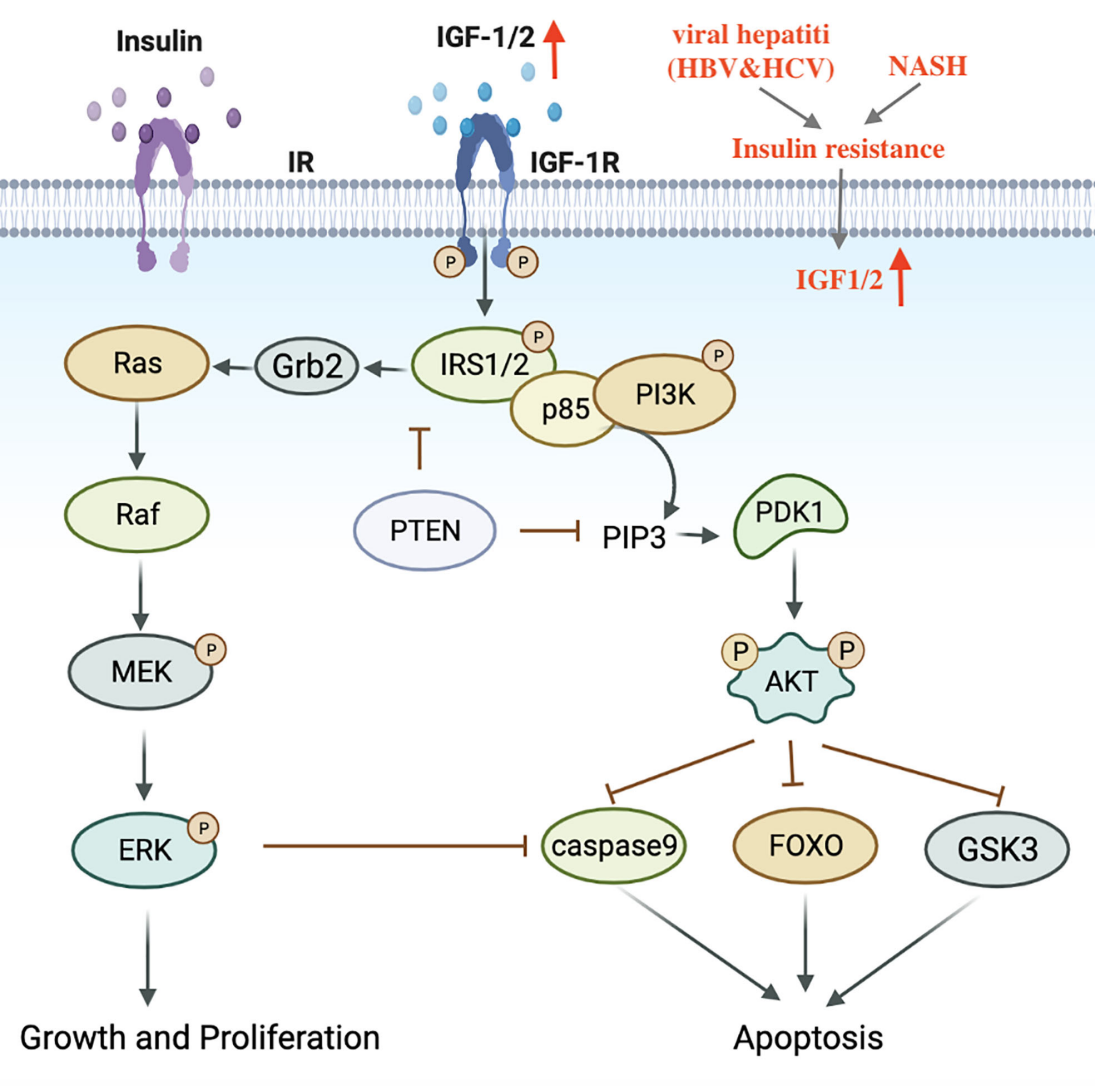
Transduction mechanism of the IGF1 signaling pathway in HCV, NASH-induced HCC. IGF, Insulin-like growth factor; IRS, Insulin receptor substrate.

Metabolic disorders, including dyslipidemia, insulin resistance and impaired blood glucose control have been identified as contributing factors to the progression of NASH (17). Insulin resistance and hyperinsulinemia can enhance the expression of IGF-1, trigger IRS-1/2 signal cascades, activate downstream PI3K-Akt and MAPK pathways, induce cell proliferation and inhibit apoptosis (105, 106) (**Figure 3**). In addition, the accumulation of excess lipids can also lead to the over-production of free fatty acids (FFA), which produce specific lipid toxicity and influence liver cell metabolism through a cascade of signals (107). It is worth mentioning that elevated iron levels have been observed in NASH patients and are considered a risk factor for HCC development (108).

In contrast to HBV viral hepatitis, HCV is commonly associated with hepatic steatosis (109). HCV core protein plays an important role in regulating lipid metabolism. Transgenic mice that express HCV core protein can develop insulin resistance, lipid accumulation in the liver, and eventually progresses to HCC (110, 111). Koike et al. revealed that HCV core protein can bind to retinol-like X receptor (RXR)-α and continuously activate peroxisome proliferator-activated receptor-α (PPAR-α), continuously activate peroxisome proliferator-activated receptor-α (PPAR-α), promote steatosis, and induce oxidative stress, eventually leading to the occurrence of liver cancer (112, 113).

Overall, hepatitis C and NASH have similar metabolic dysregulation, including hepatic steatosis, dyslipidemia, insulin resistance, and oxidative stress. But at the same time, there are differences. The metabolic dysfunction of HCV is mainly induced by the core protein, while the metabolic dysfunction of NASH is more complicated and the specific mechanisms remain to be explored. Compared with NASH, the incidence of HCV-induced HCC is higher (114).

### 5.2 Inflammatory and immunologic pathways

**Figure 4:**
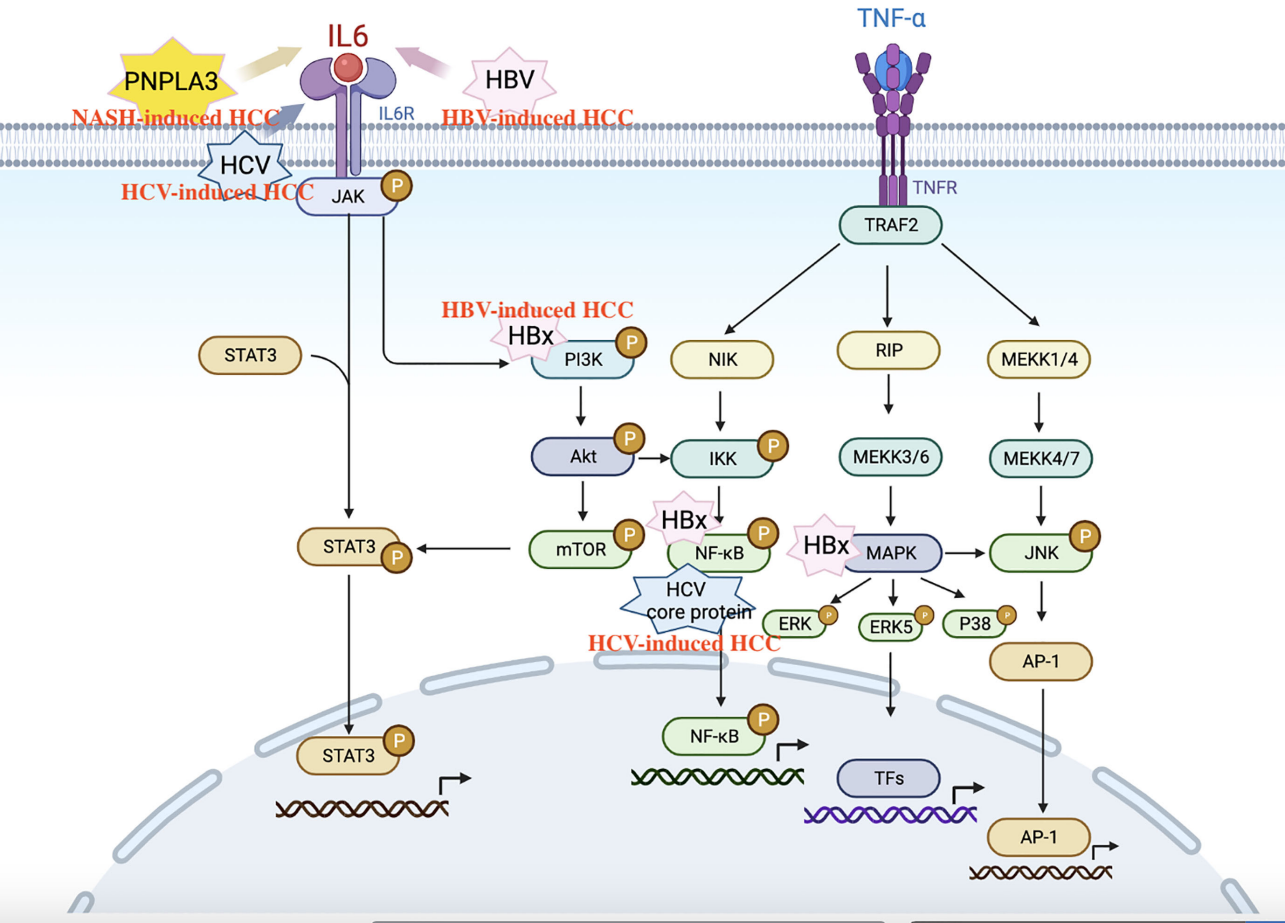
Schematic diagram of IL6 and TNF-α inflammatory pathways in HBV, HCV, NASH-induced HCC. IL-6, Interleukin-6; TNF-αα Tumor necrosis factor-alpha; HCC, Hepatocellular carcinoma.

More than 90% of HCC occurs in the context of liver inflammation. Chronic inflammation induces immune cells to secrete a variety of cytokines, including TNFα, IL-6, leptin, adiponectin, chemokines, and so on (115) (**Figure 4**). In NASH, chronic HBV, and HCV, common mechanisms driving HCC development include: the persistence of liver inflammation, immune-mediated liver injury, and ultimately up-regulated release of pro-inflammatory factors TNF-α and IL-6. TNF-α is one of the most clearly characterized pro-tumor cytokines in HCC. It can simultaneously activate NF-κB and JNK signaling pathways, promote cell survival, inhibit cell apoptosis (116–118). IL-6-mediated STAT3 activation is a major driver of hepatocyte repair and replication, which promotes HCC development (117). Studies have also shown that IL-6 expression is upregulated and STAT3 is over-activated in HCC patients (119). The chemokine CKLF1 is overexpressed in HCC and is associated with tumor stage, vascular invasion, and prognosis. It can promote the progression of HCC by activating the IL6/STAT3 pathway (120).

Specifically, PNPLA3 polymorphism can also enhance inflammatory signaling through the IL-6/STAT3 and CCL5 pathways. PNPLA3-I148M mutant mice have been reported to spontaneously develop hepatic steatosis (121). PNPLA3-I148M HCC cells can promote proliferation *via* IL6/STAT3 and enhance the activation of hepatic stellate cells by upregulating the expression of chemokine ligand 5 and collagen 1α1 (120, 122).

HBx protein has carcinogenic activity and can regulate a variety of inflammatory pathways in hepatocarcinogenesis, including MAPK, NF-κB, IL-6/STAT3, and PI3K signaling pathways (48). HBsAg can inhibit the activation of STAT3 in NK cells, leading to HBV clearance disorder and accelerate the progression of HBV hepatitis to HCC (123, 124). Meanwhile, immunosuppressive microenvironment also plays an important role in promoting tumor progression. In HBV-related HCC patients, increased peripheral blood neutrophil/lymphocyte ratio (NLR) and increased Foxp3 + Treg cell number are positively correlated with disease progression (125, 126).

As for hepatitis C, the pro-inflammatory status is maintained mainly by affecting STAT3 and NF-κB pathways (127). HCV core proteins, NS4B, and NS5B can enhance TNF-α-induced cell death by inhibiting NF-κB activation (128). HCV can also promote the transcription of STAT3 by upregulating miR-135A-5p and inhibiting its regulatory factor PTPRD, driving the progression of HCC (129).

### 5.3 Gut microflora dysregulation

Due to the tight anatomical functional crosstalk between the gut and liver, the gut microbiota and its metabolites can influence liver disease progression through the “gut-liver axis” (130). Animal models and human studies have demonstrated that increased intestinal permeability can lead to dysbiosis, resulting in the influx of pathogen-associated molecular patterns(PAMPs) and gut microbiota-derived metabolites into the liver, which further triggers hepatic inflammation and hepatocarcinogenesis (131). At the molecular level, PAMPs, such as lipopolysaccharide (LPS), enter the liver through the portal vein and are recognized by TLRs (TLR4 and TLR9) in immune cells, resulting in the production of a series of cytokines (IL, TNF, IFN) that cause liver cell damage (132). It has been reported that changes in intestinal flora and dysbiosis exist in both NASH and chronic viral hepatitis (131, 133–135).

## 6 Conclusions

In general, the progression from HBV, HCV, and NASH infection to HCC is the result of the accumulation of multiple factors and the interaction of multiple mechanisms. For HBV-induced HCC, HBV DNA integration was observed in nearly 80% of tumor tissues. HBV-induced epigenetic dysregulation (DNA methylation, Histone modification, MicroRNAs), HBV-encoded proteins (HBx, HbsAg, and core protein), and genetic variations play a central role in HCC development. For HCV-induced HCC, HCV-induced epigenetic dysregulation, HCV-encoded proteins (NS3, NS5A, NS5B, and core protein), and genetic variations can lead to hepatocarcinogenesis. For NASH-induced HCC, it is more dependent on genetic factors (PNPLA3, 17β-HSD13, TM6SF2 variant) and epigenetic dysregulation.

In addition, some of the same mechanisms also exist in the process of viral hepatitis and NASH-induced HCC, lipid metabolism disorders, persistent pro-inflammatory, immune responses, and intestinal microbiota dysbiosis are all involved and play a crucial role. Abnormal lipid accumulation, insulin resistance, oxidative stress, and other metabolic disorders are present in both hepatitis C and NASH-induced HCC. Persistent liver inflammation, immune-mediated liver damage, and upregulation release of proinflammatory cytokines TNF-α and IL-6 are present in NASH, chronic HBV, and HCV-induced HCC. Due to the presence of the gut-liver axis, intestinal microbiological changes and dysbiosis caused by viral hepatitis and NASH can further trigger hepatic inflammation and hepatocarcinogenesis through PAMPs.

In early stage HCC, the most effective treatment options are surgical resection, liver transplantation, or percutaneous local ablation. Systemic therapy with various drugs targeting the tumor microenvironment (TME) for unresectable HCC has been shown to be effective. Multi tyrosine kinase inhibitors(TKIs), such as sorafenib, lenvatinib, regorafenib, cabozantinib, and the vascular endothelial growth factor inhibitor (ramucirumab), have been widely used in clinical. Sorafenib exerts anti-tumor effects *via* inhibiting vascular endothelial growth factor receptor (VEGFR), Raf-1, B-Raf, and platelet-derived growth factor receptor (PDGFR) (136, 137). In addition to TKIs, new therapeutic strategies such as immune checkpoint inhibitors (ICIs) have also progressed in recent years (138). Two anti-PD-1 drugs, nivolumab and pembrolizumab, have been approved as second-line treatment for patients with sorafenib-refractory advanced HCC in the United States (139, 140). Even so, the prognosis of patients with advanced HCC is still not optimistic, and the prognosis of unresectable HCC remains poor. Exploring the pathogenesis of viral hepatitis and NASH-induced HCC may provide guidance for the development of new molecular therapeutic targets and therapeutic drugs. At the same time, it may also play an important role in judging the prognosis of patients and providing individualized treatment.

In general, the innovation of this paper is that we discuss viral hepatitis-induced and NASH-induced HCC together for the first time, and analyze differences and connections of them, which have not been seen in the published reviews. However, the description of the potential molecular pathogenesis in the article is not in-depth. We will focus on a certain direction to further investigate the underlying molecular mechanism in our future studies.

## Author contributions

Writing—original draft preparation: ZT, CX, PY, WW, ZL, WZ, and JD; writing—review and editing: XZ and KD. All authors contributed to the article and approved the submitted version.

## Funding

This work was supported by the Academician expert workstation of Shaanxi Province, and the National Natural Science Foundation of China (No.81870446, 82070671, 81900571, 82070681).

## Conflict of interest

The authors declare that the research was conducted in the absence of any commercial or financial relationships that could be construed as a potential conflict of interest.

## Publisher’s note

All claims expressed in this article are solely those of the authors and do not necessarily represent those of their affiliated organizations, or those of the publisher, the editors and the reviewers. Any product that may be evaluated in this article, or claim that may be made by its manufacturer, is not guaranteed or endorsed by the publisher.
